# Cytoskeleton in the Parasitic Plant *Cuscuta* During Germination and Prehaustorium Formation

**DOI:** 10.3389/fpls.2018.00794

**Published:** 2018-06-13

**Authors:** Peter Kaštier, Yuliya A. Krasylenko, Michaela Martinčová, Emmanuel Panteris, Jozef Šamaj, Alžbeta Blehová

**Affiliations:** ^1^Department of Plant Physiology, Faculty of Natural Sciences, Comenius University in Bratislava, Bratislava, Slovakia; ^2^Department of Cell Biology, Centre of the Region Haná for Biotechnological and Agricultural Research, Faculty of Science, Palacký University Olomouc, Olomouc, Czechia; ^3^Department of Cell Biology and Biotechnology, Institute of Food Biotechnology and Genomics, National Academy of Sciences of Ukraine, Kiev, Ukraine; ^4^Department of Botany, School of Biology, Aristotle University of Thessaloniki, Thessaloniki, Greece

**Keywords:** parasitic weeds, dodder, *Cuscuta*, prehaustorium, root-like structure, shoot apical meristem, microtubules, actin filaments

## Abstract

Although cytoskeleton is a driving force for cell division and growth in higher plants, there is little evidence about its components in parasitic angiosperms. Microtubules and actin filaments in cells of shoot apical meristem and root-like structure of stem holoparasites European (*C. europaea* L.) and Eastern (*C. monogyn*a Vahl.) dodders, as well as in prehaustorium, the specific organ adapted to parasitism, were visualized for the first time by immunolabeling and fluorescence microscopy. The significance of cytoskeletal elements during germination and prehaustorium formation was addressed by treatments with taxol, oryzalin, latrunculin B, cytochalasin B/D, jasplakinolide, and 2,3-butanedione monoxime. In shoot apical meristem many dividing cells were observed, in contrast to root-like structure, devoid of cell divisions. Cortical microtubules were oriented transversely and/or obliquely, while actin filaments were randomly distributed in cells of both organs. Furthermore, longitudinal cortical microtubules were present in digitate cells of prehaustorium, and transverse arrays were found in its file cells. Long and short random actin filaments were also observed in prehaustorium cells. Thus, it was shown that the cytoskeleton in dodder shoot cells is organized in a similar way to non-parasitic dicots, while cytoskeletal organization has some peculiarities in quickly senescing root-like structure and prehaustorium.

## Introduction

Parasitic plants are widespread weeds, agronomically and economically important in terms of harvest devastation. They need host plants as a source of nutrients, both organic and growth substances for their survival, and exude their metabolites into these host plants. Parasitic plants have developed various ways of attacking their hosts using a special adhesion/absorption organ called haustorium. The haustorium is able to penetrate stems, leaves or roots of the hosts. Among 4,500 flowering parasitic plants (around 1% of all angiosperm species) (Yoshida et al., 2016), dodders (*Cuscuta* spp.) are represented by approximately 200 holo- and hemiparasitic species that contain trace amounts of chlorophyll and no RUBISCO activity (Kuijt and Toth, 1976; van der Kooij et al., 2000; Těšitel, 2016). Many *Cuscuta* species comprise Class 1 designated Prohibited Noxious Weeds (Weed Seeds Order, 2016) as they cause the reduction of the host plant yields, play a role of vectors for diseases (phytoplasma, yellow diseases group, etc.) and cause toxic gastrointestinal upset danger for animals (Saric-Krsmanovic et al., 2017). *Cuscuta* natural hosts are mainly dicotyledonous plants from Brassicaceae, Leguminosae, Solanaceae, and other taxa (García et al., 2014). Formation of haustorium is a necessary first step of parasitism establishment, essential for mRNA trafficking between parasite and host xylem and/or phloem tissues (Kim and Westwood, 2015; Yoshida et al., 2016).

Dodder seedlings emerge with thread-shaped hypocotyls, using nastic movements and chemotropism for host recognition, having neither roots nor cotyledons. Later, they develop filiform climbing stems with scale-like leaves, completely dependent on a host for support, water, photosynthetic assimilates and nutrients (Těšitel, 2016). Most dodders form only rudimentary roots (root-like structures) with root apices surrounded by a circle of trichomes resembling root hairs. They become senescent by the 7th–10th day and collapse completely by the 14th–20th day post-germination, transferring the baton of growth to haustorium in a sort of developmental treadmilling essential for dodder survival (Lyshede, 1985, 1986; Sherman et al., 2008; Kaštier et al., 2017). Dodder shoots have mitotically active cells in the apex and lack mechanical tissues (Toma et al., 2005; Sherman et al., 2008). The arrangement of the xylem bundles is random (scattered type) or circular (collateral vascular bundles), which is unique for these plant species (Toma et al., 2005). The development of European (*C. europaea* L.) and Eastern (*C. monogyna* Vahl.) dodders shows a remarkable degree of plasticity, mostly due to specialized tissues such as endogenous disk-like meristems, which are essential for haustorium formation. Generation of mechanical stimulus, following initial contact with the host plant, induces cell differentiation and haustorium formation, and its subsequent penetration into the host stem. This is facilitated by the recruitment of stress-responsive and defense genes for host recognition and activity of cell wall-modifying enzymes (Srivastava et al., 1994; Vaughn, 2002, 2003).

Although the morphology and anatomy of *Cuscuta* spp. are well studied, the cellular mechanisms of the interactions between parasitic plants and their susceptible hosts are not well understood. Especially, the cytoskeleton organization remains largely unexplored. Dynamic reorganization of microtubules and actin microfilaments is crucial for plant cell division and expansion (Kost et al., 2002; Wasteneys and Ambrose, 2009; Smertenko et al., 2017) as well as for plant responses to biotic stresses (Takemoto and Hardham, 2004; de Almeida Engler et al., 2010). Cytoskeleton is involved in plant susceptibility to various pathogens and symbionts, both at the level of their attachment to the plant host (e.g., by ciliae, flagellas, exomycorrhizal mantle, etc.) and accommodation of infection/symbiotic structures (e.g., penetration pegs, appressoria, hyphae, arbuscular/rhizobial mycorrhiza coils, orchid pelotons, etc.) (Lapin and Van den Ackerveken, 2013). Although the cytoskeletal patterns in parasitic plants have not been described yet, microtubules and actin filaments are expected to be broadly involved in the immune responses (Yoder and Scholes, 2010).

Early study on *C. pentagona* L. (Sherman et al., 2008) revealed polypeptide bands at 43 and 55–56 kDa, corresponding to actin and α-tubulin, on Western blots from root and shoot protein extracts. The presence of large strands resembling actin cables on electron micrographs of *C. pentagona* L. searching hyphae was mentioned by Vaughn (2003). F-actin rearrangement during haustorium differentiation in *Striga* was described as well (Florea and Timko, 1997). However, the organization of both microtubules and actin filaments in cells of shoots, root-like structures and (pre)haustorium have not been studied before. Therefore, we aimed to visualize cytoskeleton components in different tissues of dodders–European (*C. europaea* L.) and Eastern (*C. monogyn*a Vahl.)–during the early ontogenetic stages and prehaustorium formation using immunohistochemical techniques. The knowledge about the cytoskeleton during host–parasite interactions could contribute significantly to the establishment of new efficient strategies to reduce the economic damage caused by noxious stem parasitic plants.

## Materials and Methods

### *Cuscuta* Seed Collection

Seeds of European (nettle) dodder (*C. europaea* L.) parasitizing common nettle were harvested in August 2015 in the field of Ivanka pri Dunaji, Slovak Republic. *C. europaea* seeds parasitizing also goat’s-head (*Tribulus terrestris* L.) were collected in October 2017 in the city of Thessaloniki, Pylaia, Northern Greece. *Nicotiana tabacum* L. and *Nicotiana benthamiana* Domin. were employed as the hosts in a greenhouse (Supplementary Figure S1).

Eastern dodder (*C. monogyn*a Vahl.) mature seeds were collected in the Crimean peninsula during August–September 2013–2017 from plants parasitizing European smoketree (*Cotinus coggygria* Scop.) (Besh-Tash Ridge, Kara-Dag Mountain group; Uzun-Syrt Ridge, Koktebel vicinity) and the Crimean endemic pistachio tree (*Pistacia mutica* Fisch. and C.A.Mey.) (Cape Alchak, S/SW slopes, Sudack terr.) (The Red Book of Ukraine) (Supplementary Figure S2).

Different dodder species were chosen intentionally, since *C. europaea* is a widespread dodder from the subgenus *Cuscuta* (Costea et al., 2015) with white thin climbing stems parasitizing mostly herbaceous hosts (Supplementary Figure S1), while *C. monogyna* is taxonomically distant species from subgenus *Monogynella* (one style gynoecium), having thick stems attributed mostly to bushes and even trees (Supplementary Figure S2). Evident interspecies differences are in seed morphology, stem and root-like structure diameter as well in the color of the seedlings and waxy cuticle covering epidermis.

### *Cuscuta in Vitro* Cultivation

*Cuscuta* seeds stored at 4°C underwent scarification in concentrated sulfuric acid (H_2_SO_4_) for 1 h with further sterilization in 4.7% w/v sodium hypochlorite solution supplemented with 0.1% (v/v) Triton-X100, short-spin vortexing, immersion into 70% ethanol for 5 s and thorough rinsing in sterile distilled water. Sterile seeds were placed on the surface of half-strength MS medium composed of 3.5 g/L MS salts, 20 g/L sucrose, 0.1 g/L asparagine, 0.1 g/L inositol and 0.02785 g/L FeSO_4_⋅7H_2_O, pH 5.8 (Murashige and Skoog, 1962) solidified by 0.6% (w/v) agarose (Sigma-Aldrich) or 0.2% (w/v) Gellan Gum (Nacalai Tesque). Petri dishes were placed horizontally in Phytotron under controlled environmental conditions: 16 h light/8 h dark, 22°C, and 120–150 μmol/m^2^s light intensity. At the same time, seeds of both dodders were germinated on wet cotton in non-aseptic experiments. It has to be noticed that *C. monogyna* was introduced *in vitro* for the first time and has shown 100% germination rate, while in *C. europaea* it was around 70%.

### Cytoskeleton Drugs Treatment

Seedlings were cultivated in Petri dishes on solid medium supplemented with taxol (10 μM), oryzalin (10 μM), latrunculin B (1–10 μM), cytochalasin B and D (10–100 μM), jasplakinolide (1 μM), and 2,3-butanedione monoxime (BDM) (20 mM) (Sigma-Aldrich). MS medium without any additives was the main control and DMSO-supplemented medium (solvent for cytoskeleton-disrupting drugs) was used at 0.01% (v/v) concentration as a mock control. Seedlings were taken at 4th and 7th days post-germination for the measurement of growth, tracking of the morphological changes and visualization of cytoskeleton. For the studies of the seedling length and morphology, 4th–7th day-old plants were captured by digital camera Canon EOS600D (Canon, Japan) and Axio Zoom.V16 Stereo Zoom system (Carl Zeiss, Germany) in bright field illumination (objective lenses PlanApo Z 1.5x, FWD = 30 mm), and processed in ImageJ software using Fiji macros (an open platform for scientific image analysis^1^). The lengths of the seedlings (*n* = 30 for each treatment, three biological repetitions) are presented as mean ± SE.

To study effects of cytoskeletal drugs on prehaustorium formation the root-like structures (4–5 cm long) of 5-day-old *C. europaea* seedlings were dipped into the Eppendorf tubes with liquid ½ MS medium supplemented with the drugs in concentrations mentioned above. Tubes were thoroughly sealed with Parafilm to prevent evaporation and placed in close proximity to the compatible host *Nicotiana benthamiana* for 48 h.

### Whole-Mount α-Tubulin Immunolabeling at Early Developmental Stages

*Cuscuta* root-like structure and shoot apex at 1st–7th day after germination were hand cut and fixed in 4% (w/v) paraformaldehyde in PEM buffer (50 mM PIPES, 5 mM EGTA, 5 mM MgSO_4_, pH 6.8) for 1 h. After washing in PEM, cell walls were digested by a cocktail of 3% (w/v) macerozyme R10 and 3% (w/v) cellulase R10 (Duchefa) in PEM at room temperature for 1.5 h (modified according to Šamajová et al., 2014). The next steps were the incubation of the samples in absolute methanol (at -20°C) for 30 min and extraction with 5% (v/v) DMSO + 1% (v/v) Triton X-100 in PBS at room temperature for 1 h. Samples were subsequently incubated overnight with rat anti-α-tubulin antibody (YOL 1/34, Serotec) diluted 1:40 in PBS. Following PBS washing, the cells were incubated overnight with FITC-anti-rat antibody (Invitrogen) diluted 1:40 in the same buffer. DNA was counterstained with 250 μg/ml 4,6-diamidino-2-phenylindole (DAPI, Sigma) in PBS for 10 min and after final washing the specimens were mounted in an antifade solution [0.5% (w/v) p-phenylenediamine in 70% (v/v) glycerol in PBS or 1M Tris-HCl, pH 8.0] or in the commercial antifade VECTASHIELD^TM^ (Vector Laboratories).

### F-Actin Staining at Early Developmental Stages

Actin filaments in the cells of shoots and root-like-structures were revealed by Atto 488 (Sigma-Aldrich)- or Dy-Light 554 (Cell Signaling)-conjugated phalloidin staining, according to Panteris et al. (2006) protocol with some modifications. Dodder seedlings (1st–7th day after germination) were incubated for actin filament stabilization in 300 μM m-maleimidobenzoyl-*N*-hydroxysuccinimide ester (MBS), prepared from a 300 mM DMSO stock, in PEM with 0.1% (v/v) Triton X-100 in darkness for 30 min. Subsequently, they were fixed in 4% (w/v) PFA + 5% (v/v) DMSO and 1:400 phalloidin, washed thoroughly and extracted with 5% (v/v) DMSO + 1% (v/v) Triton X-100 + 1:400 phalloidin during 60 min. The staining itself was performed with 1:40 phalloidin in PBS buffer at 37°C for 2 h. The following steps were performed according to the procedure described above for the microtubules.

### Co-immunolabeling of Microtubules and Actin Filaments Using Steedman’s Wax Technique

Steedman’s wax embedding of dodder prehaustoria for actin and tubulin immunolocalization was performed according to Vitha et al. (2000). The samples were fixed as for the whole-mount technique. A cold absolute methanol (-20°C) step was applied for 30 min and then the samples were dehydrated in ethanol/PEM with a gradual increase of ethanol concentration from 10% to 30%, 50%, 70%, 90% up to twice 100% (v/v); each step at room temperature for 30 min. Dehydrated samples were embedded into Steedman’s wax, which was prepared by dissolving PEG-400 DS (polyethylene glycol 400 distearate, Sigma-Aldrich) and 1-hexadecanol (Sigma-Aldrich) in 9:1 ratio. Samples were cut using a rotary microtome (HM 325, Thermo Scientific) to obtain 40 μm-thick sections. Rehydrated tissue sections (in ethanol series) were extracted with 5% (v/v) DMSO and 1% (v/v) Triton X-100 in PBS for 1 h, then incubated overnight at room temperature with rat anti-α-tubulin antibody (YOL 1/34, Serotec) diluted 1:40 in PBS and with rabbit anti-actin antibody (AS16, Agrisera) diluted 1:200 in PBS. After washing with PBS, the samples were incubated overnight with FITC-anti-rat (diluted 1:40) and Alexa-Fluor 555 (Invitrogen, diluted 1:300) antibodies in PBS. The final steps were performed according to the procedure described for the microtubules.

All fluorescent specimens were examined under confocal laser scanning microscope (CLSM) LSM710 or LSM780 (Carl Zeiss, Jena, Germany) with the appropriate laser settings and following Zeiss software instructions.

## Results

### Cytoskeleton Organization in *Cuscuta* Seedlings

**FIGURE 1:**
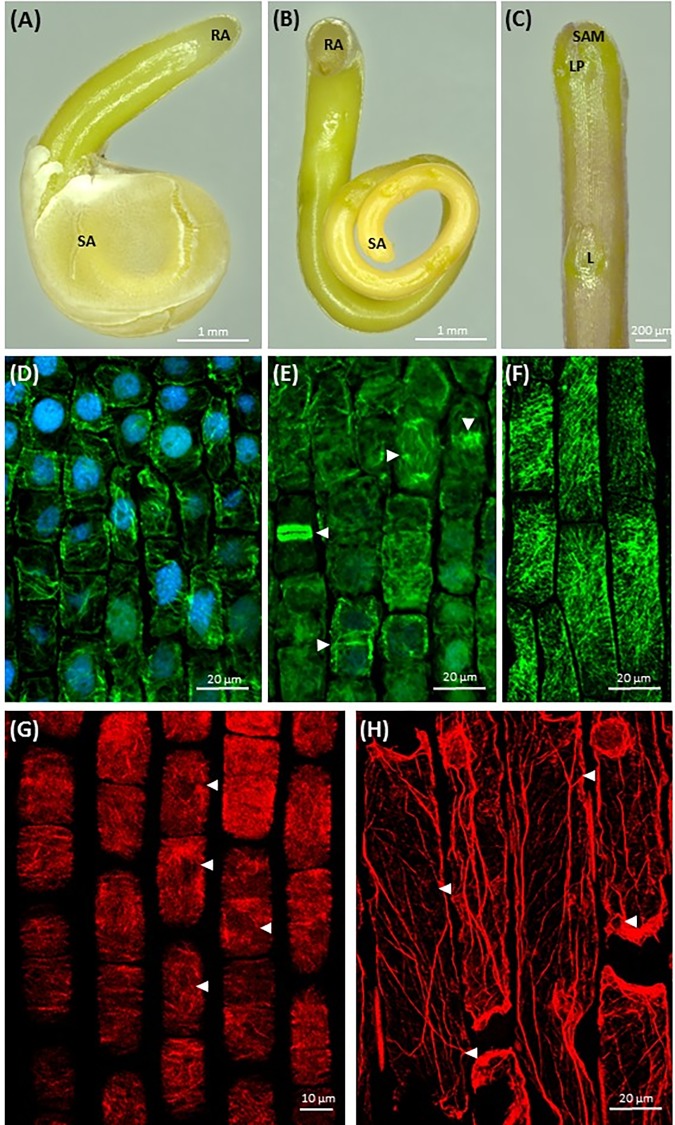
Morphology of dodder seedlings and maximum intensity projection images of cytoskeleton arrays in shoot apex cells: **(A,B)** bipolar filiform seedling of *C. monogyna* devoid of cotyledons, spirally coiled with a tapered shoot apex (SA) covered with testa’s remnants or released, and a blunt radicular end (RA), 2nd day post-germination; **(C)** shoot apex of *C. monogyna* with scale-like leaf (L), leaf primordia (LP), and shoot apical meristem (SAM); 4th day; **(D)** cortical and endoplasmic microtubules (green) in *C. europaea* shoot apical meristem cells, DAPI-stained nuclei (blue), 7th day; **(E)** endoplasmic microtubules, mitotic spindles, phragmoplast and two daughter cells after cytokinesis (arrowheads) in *C. europaea* shoot apical meristem, DAPI-stained nuclei, 7th day; **(F)** cortical microtubules in *C. monogyna* shoot cortical cells; **(G)** actin filaments in *C. europaea* shoot apical meristem cells, 7th day; **(H)** actin filaments in *C. monogyna* shoot cortical cells, 7th day.

Germinating bipolar seedlings of *C. europaea* and *C. monogyna* have active shoot apical meristem on the shoot terminus, covered with endosperm and testa remnants (**Figures 1A–C**, Supplementary Figure S3), and root apex on massive semi-circular anchorage root-like structure (**Figure 1B**). After dodder germination, almost two-fold coiled embryonic axis aligns along the stem of host plant. Leaf primordium (LP) is located at the close proximity of shoot apex (**Figure 1C**). The fast growth of dodder seedlings is maintained by abundant microtubules (**Figure 1D**) in shoot apical meristem cells and their high meristematic activity by the rearrangement between cortical and mitotic arrays (**Figure 1E**). Shoot apical meristem cells of 7-day-old seedlings of both dodders are predominantly isodiametric, with centrally positioned large nuclei surrounded with a dense network of endoplasmic microtubules (**Figure 1D**). Cortical microtubules in cortical cells of rapidly elongating dodder shoots were oriented mostly transversely, while in some cells their alignment can be also oblique (**Figure 1F**).

Besides microtubules, shoot apical meristem cells had a network of actin filaments represented by perinuclear F-actin bundles (**Figure 1G**, arrowheads). The most commonly observed patterns of cortical actin filament alignment in shoot apical meristem cells were transverse, radial and/or random (**Figure 1G**). In turn, longitudinally oriented actin cables were uniformly distributed throughout the cytoplasm of cortical cells of *Cuscuta* shoot (**Figure 1H**). Some of these long F-actin cables traversed the cytoplasm toward its cortical layer (**Figure 1H**, arrowheads).

**FIGURE 2:**
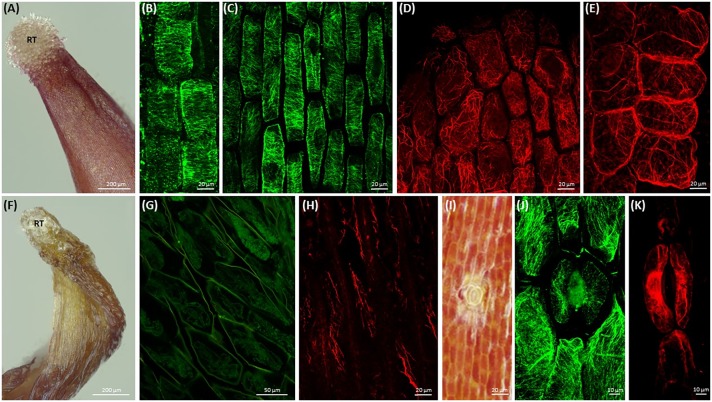
Morphology of root-like structures of dodder seedlings and maximum intensity projection images of cytoskeleton array alignment: **(A)** root-like structure of *C. monogyna* with the corolla of terminal root hair-like trichomes (RT); 4th day post-germination; **(B)** cortical microtubules in cortical cells of radicle, 1st day; **(C)** cortical microtubules in root-like structure cortical cells, middle area, 3rd day; **(D,E)** actin filaments in root apex cells, 3rd day; **(F)** degrading *C. monogyna* root-like structure with the wilted terminal root hair-like trichomes; 7th day; **(G,H)** depolymerized cortical microtubules **(G)** and actin filaments **(H)** in plasmolyzed root-like structure cells, 7th day; **(I)** anomocytic stoma surrounded by six subsidiary cells and epidermal cells of *C. monogyna* root-like structure, 4th day; **(J,K)** cortical and endoplasmic microtubules **(J)** and actin filaments **(K)** in stomatal and subsidiary cells of *C. monogyna* root-like structure, 2nd day.

Root-like structure of both *Cuscuta* species ends with a corolla of root hair-like trichomes (**Figure 2A** and Supplementary Figure S3), ending up wilted and plasmolyzed at the 7th day post-germination (**Figure 2F**).

**FIGURE 3:**
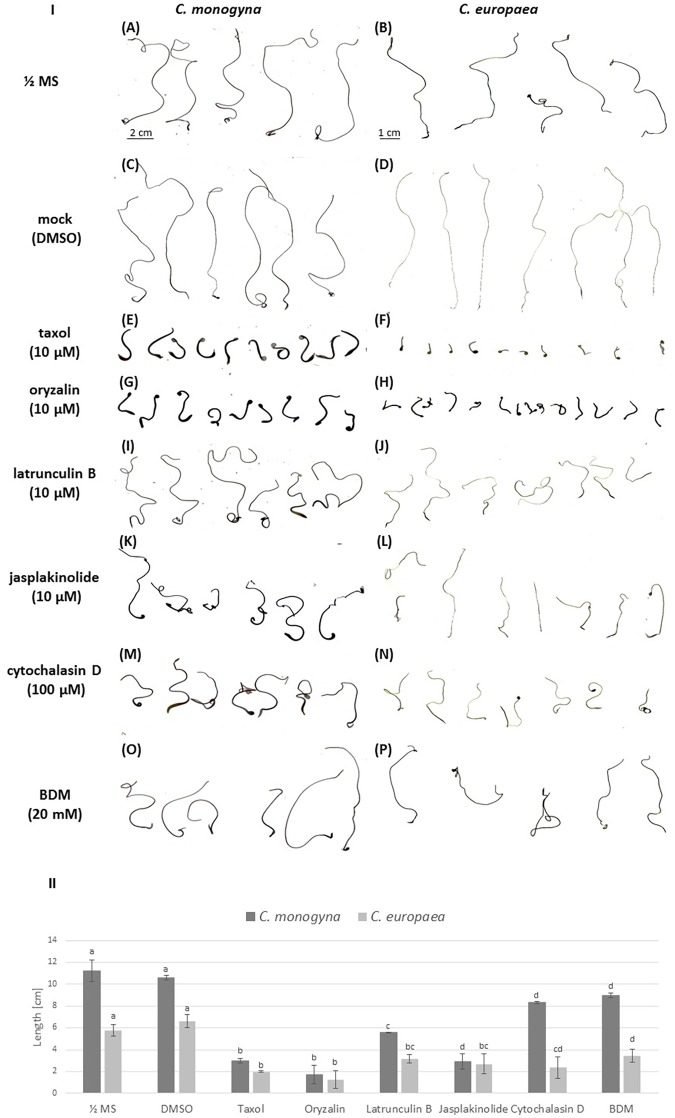
**(I)** The inhibition of 7-day-old *C. monogyna* and *C. europaea* seedlings by cytoskeletal drugs: scanned seedlings of *C. monogyna* and *C. europaea*. **(A,B)** ½ MS medium; **(C,D)** mock control (0.001% DMSO in MS); **(E,F)** taxol (10 μM); **(G,H)** oryzalin (10 μM); **(I,J)** latrunculin B (10 μM); **(K,L)** jasplakinolide (10 μM); **(M,N)** cytochalasin D (100 μM); **(O,P)** BDM (20 mM). **(II)** Graphical presentation of seedling growth inhibition by cytoskeletal drugs. The lengths of the seedlings (*n* = 30 for each treatment, three biological repetitions) are presented as mean ± SE. The letter above the column indicates significant difference (Tukey HSD, *P* < 0.05).

Furthermore, the highly enlarged and vacuolated cells of massive bulbous *Cuscuta* root-like structures were characterized by residual or absent mitotic activity, since no preprophase bands, mitotic spindles and phragmoplasts were revealed in root apex cells even at the 1st (**Figure 2B**) and 3rd (**Figure 2C**) days post-germination.

Despite the absence of mitotic microtubule arrays, transverse cortical arrays were present in the root apex cells (**Figure 2C**) before the anchorage root starts to degrade at 5th-7th day post-germination (**Figure 2F**). However, starting from the 3rd day, microtubules gradually lost their ordered alignment, became more disorganized, fragmented, and finally depolymerized during programmed developmental changes up to 7th day post-germination (**Figure 2G**).

Simultaneously, at early stages of root-like structure development (1st-4th day post-germination) the cytoplasm of root apex and cortical cells included a dense network of F-actin bundles (**Figures 2D,E**). Actin filaments were forming a branched net in the apex (**Figure 2D**). Within the next few days up to 7th day post-germination, the actin network was degraded and fragmented as root-like structure cells underwent programmed plasmolysis (**Figure 2H**).

Open anomocytic stomata (non-secretory stomatiferous protuberances) surrounded by six epidermal subsidiary cells were present in shoots, but also in root-like structures (up to five per *C. monogyna* and *C. europaea* radicular ends) (**Figure 2I** and Supplementary Figure S3). Radially organized cortical microtubules were found in guard cells, and random ones in subsidiary cells (**Figure 2J**). Endoplasmic F-actin cables aligned along the stomatal aperture, also surrounding the guard cell nuclei (**Figure 2K**).

### Changes in the Growth and Morphology of *Cuscuta* Seedlings Caused by Cytoskeletal Drugs

A set of cytoskeletal drugs affecting diverse cytoskeletal components was used in further experiments on germinating *Cuscuta* seedlings. Microtubules were stabilized by taxol (paclitaxel) (Baluška et al., 1997; Wani and Horwitz, 2014) and depolymerized or prevented from further polymerization by the dinitroaniline herbicide oryzalin (Morejohn et al., 1987). Actin filament network was compromised by latrunculin B (Spector et al., 1999; Baluška et al., 2001), which binds G-actin monomers and prevents their polymerization (Gibbon et al., 1999), or by cytochalasin D. Jasplakinolide was used to induce actin polymerization and/or stabilization of the pre-existing actin filaments (Šamaj et al., 2002; Holzinger and Blaas, 2016). The interactions between F-actin and myosin were disturbed by BDM, a general inhibitor of myosin ATP-ases of eukaryotic cells (Šamaj et al., 2000). The approach of the 48 h-long soaking of just germinated dodder seedlings in liquid ½ MS medium supplemented with these cytoskeletal drugs (early growth response) was not as efficient (data not shown) as the cultivation of dodder seedlings on solid medium supplemented with cytoskeleton drugs (late growth responses) up to 7th–10th day after germination. Thus, a severe retardation of seedling growth in response to taxol and oryzalin was observed, while latrunculin B, cytochalasin D, jasplakinolide and BDM caused moderate inhibition, as compared to the untreated seedlings and mock control with DMSO (**Figure 3**).

**FIGURE 4:**
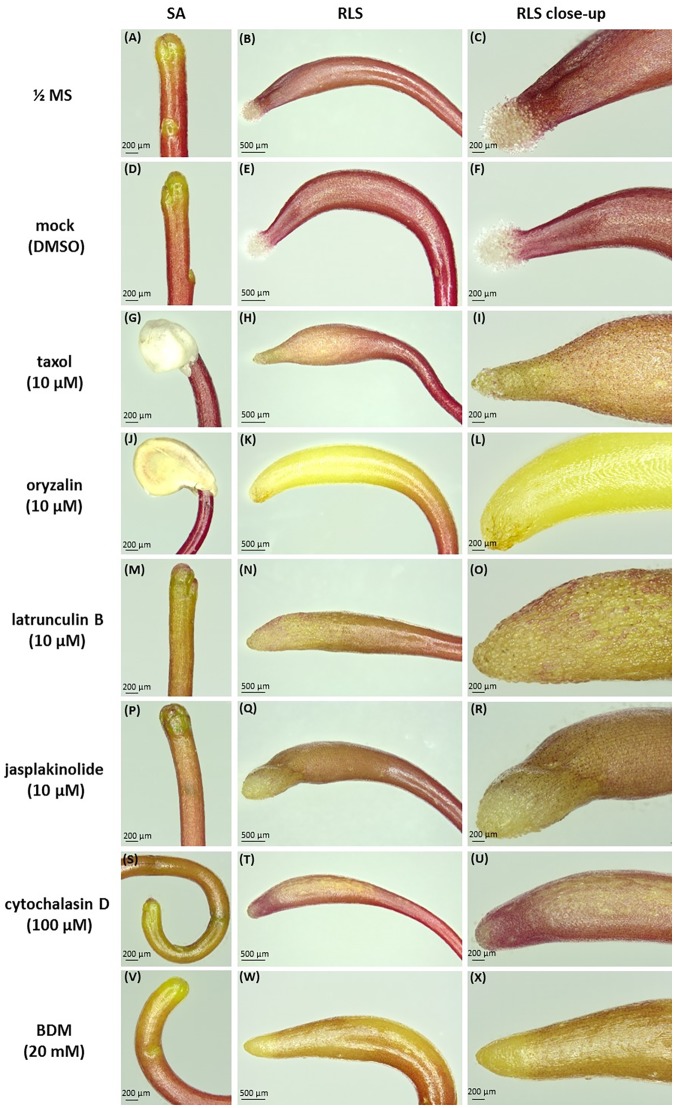
Effects of cytoskeletal drugs on 4-day-old *C. monogyna* seedling morphology. **(A–C)** ½ MS medium; **(D–F)** mock control (0.001% DMSO in MS); **(G–I)** taxol (10 μM); **(J–L)** oryzalin (10 μM); **(M–O)** latrunculin B (10 μM); **(P–R)** jasplakinolide (10 μM); **(S–U)** cytochalasin D (100 μM); **(V–X)** BDM (20 mM). SA, shoot apex; RLS, root-like structure.

Concerning the effects of the drugs on dodder morphology, most of them caused anisotropic club-like swelling of both shoot apex and root-like structure, and also the degradation of the root-hair like trichomes (**Figure 4** and Supplementary Figure S4). Besides the pronounced swelling of both shoot and root-like structure cells (**Figures 4H,I,K,L**), taxol and oryzalin treatment prevented seed coat removal from the shoot (**Figures 4G,J**), since the shoot apex remained coiled inside the seed coat even at the 7th day post-germination (Supplementary Figures S4, S5). Oryzalin treatment bleached out the dark-red pigmentation of root-like structures to a light yellow one (**Figures 4K,L**). It has to be noticed that the swelling of root-like structures after jasplakinolide, cytochalasin D and BDM treatments (**Figures 4R,U,X**) was not as pronounced as that induced by taxol, oryzalin, and latrunculin B (**Figures 4I,L,O**).

The hooked apices of most root-like structures were wilted on the 7th day post-germination (Supplementary Figures S4, S5B,C), except the taxol- and oryzalin-treated ones (Supplementary Figures S4, S5F–L). In the latter case the progression of physiological developmental changes connected with root-like structure degradation, including the typical invaginations of its tissue and wilting of root hairs-like trichomes starting from 4th day post-germination, were partially blocked (Supplementary Figures S3, S4, S5C).

### Microtubules and Actin Filaments in Dodder Seedlings Affected by Cytoskeletal Drugs

**FIGURE 5:**
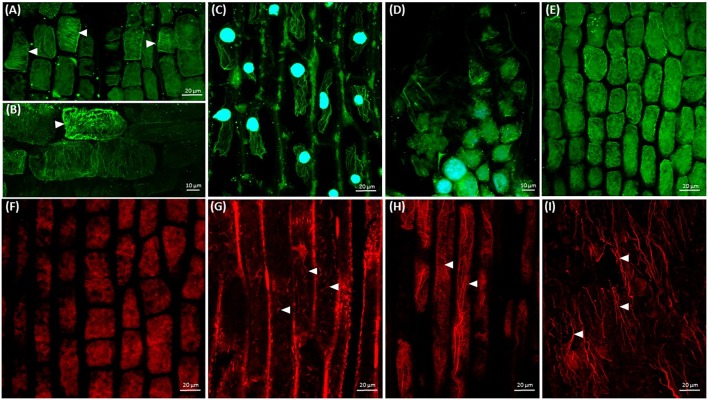
Arrays of cortical microtubules (green) and actin filaments (red) in *Cuscuta* cells. Nuclei (blue) are stained with DAPI. **(A,B)** Taxol (10 μM) stabilized cortical microtubules (arrowheads) in shoot cells; **(C)** taxol (10 μM) stabilized microtubules in root-like structure cells, 7th day; **(D)** oryzalin (10 μM) induced disruption of microtubules in shoot apical meristem cells; **(E)** oryzalin (10 μM) induced disruption of microtubules in shoot cortical cells; **(F)** cytochalasin B (100 μM) induced disruption of actin filaments in shoot apical meristem cells; **(G)** cytochalasin B (100 μM) induced fragmentation of actin filaments in shoot cortical cells; **(H)** longitudinally oriented F-actin cables and cortical transverse actin arrays in shoot cortical cells after BDM (20 mM) treatment; **(I)** disturbed organization of F-actin cables in root-like structure plasmolyzed cells after BDM (20 mM) treatment, 7th day.

Since the observed changes in dodder growth and development are based on the disturbance of microtubules and actin filaments, the next step was to study their organization after the treatment with cytoskeletal drugs. Cortical microtubules exhibited high transverse and partially longitudinal configurations in shoot cortical cells of taxol-treated seedlings (**Figures 5A,B**).

Moreover, cortical microtubule arrays (arrowheads) were partially stabilized in shoot apical meristem affected by taxol (**Figure 5A**) as compared to the control (**Figures 2B,C**). It is noteworthy that in many physiologically plasmolyzed cells of root-like structure microtubules stabilized by taxol were still present (**Figure 5C**), in contrast to the untreated seedlings (**Figure 2G**), at the 7th day post-germination. Numerous plasmolyzed cells devoid of microtubules were observed as well. At the same time, oryzalin effect was more pronounced in epidermal cells of root-like structure, where no microtubules were observed (data not shown), while in shoot apical meristem and shoot cortical cells the microtubules were more resistant, though randomized, fragmented and/or partially degraded (**Figures 5D,E**).

As for the actin cytoskeleton, actin filaments were depolymerized in shoot apical meristem cells after cytochalasin B treatment (**Figure 5F**), while in shoot cells they persisted though appeared fragmented (**Figure 5G**). The architecture of F-actin in shoot cells was not dramatically compromised by BDM, since fine cortical transverse filaments coexisted with thick longitudinal bundles (**Figure 5H**), comparable to actin filaments alignment in shoot cells of untreated seedlings (**Figure 1H**). The residual actin bundles oriented mostly longitudinally were revealed in plasmolyzed cells of root-like structure at 7th day post-germination (**Figure 5I**).

**FIGURE 6:**
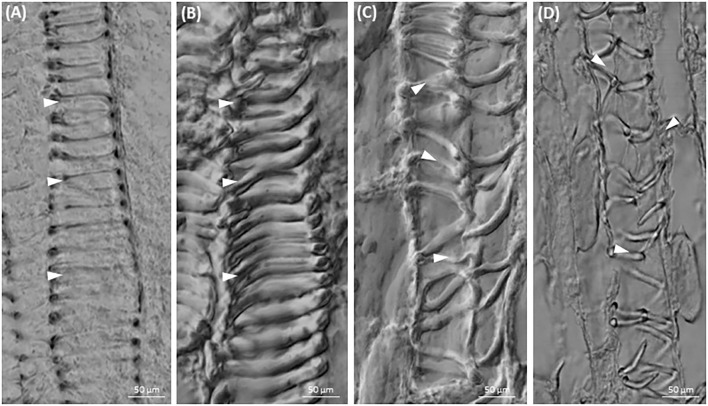
Xylogenesis in *C. europaea* shoots: **(A,B)** xylem vessels in untreated seedlings (½ MS, mock); **(C)** irregularly thickened cell walls in cells of latrunculin B- **(C)** and taxol- **(D)** treated seedlings (both in 10 μM concentration). Thickened xylem elements are indicated by arrowheads.

Since the cytoskeleton coordinates the morphogenesis of vessels, the effect of cytoskeletal drugs on xylem biogenesis was also studied. Differentiated xylem elements had regularly spaced annular wall thickenings in the control (**Figures 6A,B**), while after treatment with taxol or latrunculin B the xylem cell wall thickenings were irregularly arranged (**Figures 6C,D**).

### Cytoskeleton Organization in Different Cells of Dodder Prehaustorium

**FIGURE 7:**
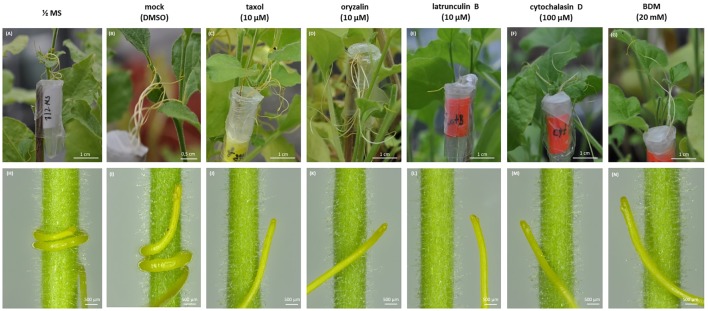
Effects of cytoskeletal drugs on prehaustorium formation on 5-day-old *C. europaea* seedlings and *Nicotiana benthamiana* as a host. **(A,H)** ½ MS medium; **(B,I)** mock (0.001% DMSO in ½ MS); **(C,J)** taxol (10 μM); **(D,K)** oryzalin (10 μM); **(E,L)** latrunculin B (10 μM); **(F,M)** cytochalasin D (100 μM); **(G,N)** BDM (20 mM).

Prehaustorium development of *C. europaea* on its susceptible host *Nicotiana benthamiana* was disturbed by cytoskeletal drugs (**Figures 7C–G,J–N**), while in untreated seedlings several prehaustorium initials were formed at the places of contact with the host stem within first 24 h (**Figures 7H,I**). Shoot apices of seedlings compromised by cytoskeleton-disrupting drugs (**Figures 7H–N**) were unable to entwine around the host by 360° double twist as compared to controls (**Figures 7A,B,H,I**). Moreover, taxol and oryzalin caused swelling of shoot apex and inhibition of its growth (**Figures 7J,K**), while the seedlings treated with the actin-disrupting drugs (**Figures 7L,M**) and BDM were longer and less swollen (**Figure 7N**) as compared to the tubulin-targeted drugs, which was in accordance with the inhibition of seedling growth during germination at earlier developmental stages.

**FIGURE 8:**
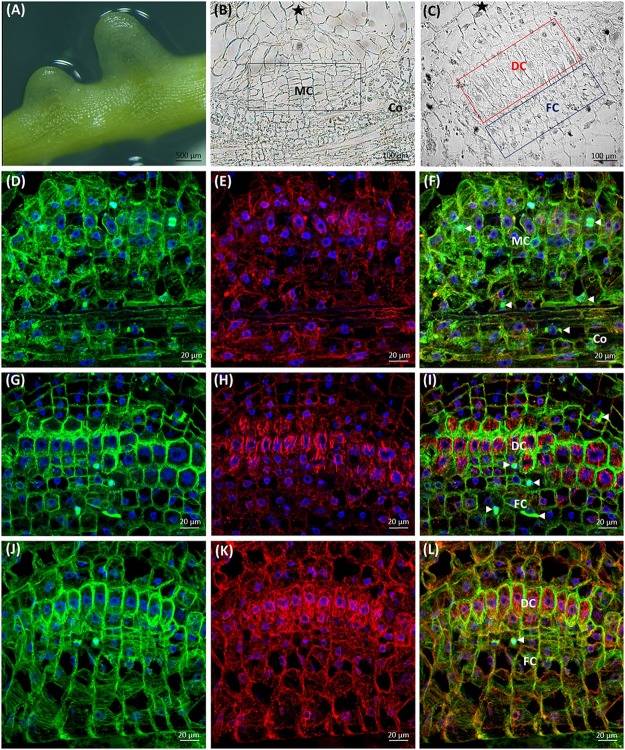
Development of *C. europaea* prehaustorium initiated by a first contact with *Nicotiana benthamiana* as a susceptible host: **(A)** general morphology; **(B)** cross-section of prehaustorial bulge: meristematic centre (MC) and the cortex (Co) cells; **(C)** significantly extended distal digitate cells (DC) and the proximal compact file cells (FC). The sites of contact with the host are marked by asterisks (^∗^). Median longitudinal sections of *C. europaea* prehaustorium depicting immunolabeled arrays of microtubules (**D,G,J**, green), actin filaments (**E,H,K**, red), and their combination (**F,I,L**, merge). Nuclei are stained blue with DAPI. Dividing cells are marked by arrowheads (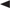): **(D–F)** meristematic centre (MC) of prehaustorium located close to xylem vessels in cortex (Co) cells, early developmental stages; **(G–I)** file (FC) and digitate (DC) cells composing the essential part of prehaustorium; **(J–L)** significantly prolonged DC and still dividing FC are present in late developmental stages.

The first contact of dodder with the susceptible host stem triggers the development of prehaustorium from the cortical cells of *Cuscuta* shoot (**Figure 8A**), preceding the formation of the fully functional haustorium (Supplementary Figures S1D, S2B,C) that connects the xylem/phloem of host and parasite. A cluster of actively dividing meristematic cells located in the close proximity to dodder’s vascular bundles is followed by a layer of mitotically active file cells and elongating digitate cells with prominent nuclei (**Figures 8B,C**). Arrays of cortical and mitotic (spindles and phragmoplasts) microtubules were present in meristematic cells, file cells and cells above the digitate ones (**Figures 8D,F,G,I**).

Subsequently, in elongating cells of prehaustorium cortical microtubules were oriented parallel to vascular bundles (**Figures 8D,F**), while at late developmental stages they gained transverse orientation (**Figures 8G,I**). In turn, in the file cells the transverse alignment of cortical microtubules persisted (**Figures 8J,L**). A network of randomly oriented actin filaments was also observed in meristematic cells of prehaustorium (**Figures 8E,F**). In addition, actin filaments formed perinuclear scaffolding arrays around centrally positioned nuclei, predominantly in digitate cells (**Figures 8H,I,K,L**).

At the time of disk-like meristem formation (**Figure 8B**), the epidermal cells of prehaustorium at the site of contact with the host start to elongate toward its stem (Supplementary Figure S6). In the zone, where the dodder shoot does not contact with the host, epidermal cells preserve the initial organization both of microtubules and actin filaments typical for the elongating cells in the growth axis of dodder shoot.

**FIGURE 9:**
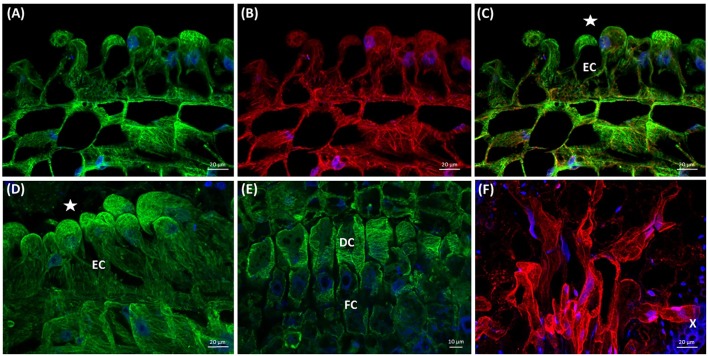
Arrays of cortical microtubules (**A,D,E** green), actin filaments (**B,F** red), and co-alignment (**C** merge) in median longitudinal sections of *C. europaea* prehaustoria **(A–E)** and searching hyphae **(F)**. Nuclei are stained blue with DAPI. Host xylem (X) bundles exhibit blue autofluorescence. The sites of contact with the host are marked by asterisks (^∗^). **(A–D)** Epidermal cells (EC) in the contact zone with the suitable host switching from their diffuse expansion to directed tip growth; **(D)** significant elongation of epidermal cells (EC) toward the host; **(E)** transversely oriented cortical microtubules in digitate (DC) and file (FC) cells during prehaustorium development; **(F)** searching hyphae that penetrated the host stem are actively growing in search of the connection with the host xylem (X).

During the elongation of epidermal cells toward the host, their nuclei were polarly located close to the cell tip, and both microtubules and actin filaments were oriented longitudinally (**Figures 9A–D**). This was in sharp contrast to the transverse alignment of microtubules in digitate cells of developing prehaustorium (**Figure 9E**). After the attachment to the host, prehaustorium evolves into the lower haustorium, which penetrates the host by formation of a fissure inside the host tissues. Epidermal cells of the lower haustorium (now called “searching hyphae”) exhibit extensive tip growth to establish contact with the host (**Figure 9F**). Such hyphae develop into a hand-like structure with grasping finger-shaped protrusions inside the host stem (**Figure 9F**) differentiating later to the xylem or phloem hyphae fully connected with the host. Abundant F-actin arrays were found in these developing searching hyphae (**Figure 9F**).

## Discussion

### The Cytoskeleton in the Unique Dodder Root-Like Structure

Although *C. europaea* and *C. monogyna* belong to different subgenera (*Cuscuta* and *Monogynella*, respectively) (García et al., 2014), their histology shares many structural similarities. Dodder shoot plasticity is ensured by the presence of endogenous disk-like meristem, necessary for the development of haustoria, and functionally active apical meristem (Svubova et al., 2017) important for the search of the potential host by chemoreception. The shoot apical meristem of both *C. monogyna* and *C. europaea* is characterized by cells with relatively conspicuous nuclei; numerous dividing cells with mitotic microtubule arrays exist, together with interphase cells with a dense network of transverse cortical microtubules (**Figure 1**). It was found previously that the contractile actin bundles with the phosphorylated myosin form a highly ordered, dynamic, and scaffolding perinuclear structure in various eukaryotic cells (Khatau et al., 2009). Shoot apex contains many dividing cells in contrast to root-like structure, where mitoses seem to be absent (Kaštier et al., 2017). These results are consistent with the findings of Sherman et al. (2008) that α- and β-tubulins were easily detectable in extracts of *C. pentagona* shoot tissues. Actin bands were detected in Western blots of *C. pentagona* protein extracts (Sherman et al., 2008), consistently with our results showing F-actin strands and perinuclear arrays in shoot apical meristem and shoot epidermal cells of both *C. monogyna* and *C. europaea* (**Figures 1D,E**).

The rudimentary (anchorage) root, also called root-like structure, reveals many differences both in anatomy and cytoskeleton organization from the primary roots of non-parasitic dicots. It lacks the typical developmental zones for dicot roots (Dolan et al., 1993), while irregular root hair-like trichomes are located at the very root tip rather than in the differentiation zone (Sherman et al., 2008). Unlike roots of dicotyledonous plants, dodders’ root-like structure does not have a functional apical meristem, Caspary bands and pericycle, while their protoxylem completely lack the secondary wall thickenings (Sherman et al., 2008). In agreement with this we did not find any cell divisions in root-like structures of 7-day-old seedlings. Root-like structure can be of shoot origin (Sherman et al., 2008), which is also supported by the presence of stomata having radial organization of cortical microtubules and longitudinal arrays of actin filaments, similar to those in non-parasitic dicot aerial organs (Galatis and Apostolakos, 2004).

At early developmental stages, transverse arrays of cortical microtubules were revealed in *C. monogyna* root-like structure cells. These arrays can support cell elongation during the first three days post-germination. After that elongation can be reduced because of the depolymerization of cortical microtubules. This corresponds well with low α-tubulin abundance in *C. pentagona* root-like structures, compared to the shoot tissue of dodder or roots of other dicots (Sherman et al., 2008). The terminal tuberous region of both *C. monogyna* and *C. europaea* root-like structures starts to deplete in several days, usually after 5th–6th day post-germination, and plays a role of nutritional reserve for the rapidly growing shoot (Sherman et al., 2008). Indeed, microtubules gradually lost their ordered alignment and became disorganized. After the 7th day post-germination only F-actin arrays were present in plasmolyzing cells of this temporary structure. This is again consistent with Western blot analysis showing a substantially higher abundance of actin, as compared to α-tubulin, in *C. pentagona* root-like structure cells (Sherman et al., 2008).

Thus, our results support an idea that the cells of root-like structure appear to undergo the process of aging accompanied by an early degradation of the microtubules (Sherman et al., 2008).

### Xylem Differentiation in Dodder Seedlings

Cytoskeleton components are involved in xylem vessel formation in higher plants (Fukuda and Kobayashi, 1989). Transverse alignment of cortical microtubules determines the patterns of thickened secondary cell wall deposition in later stages of xylem differentiation, which can be spiral, reticulate, and/or pitted (Oda et al., 2005). Microtubule-associated proteins (MAPs) such as MAP65, MAP70-5, MICROTUBULE DEPLETION DOMAIN 1 (MIDD1) and CORD1 (CORTICAL MICROTUBULE DISORDERING 1) are believed to regulate secondary cell wall patterning in xylem vessel cells (Mao et al., 2006; Pesquet et al., 2010; Sasaki et al., 2017). Cortical microtubules also play an important role during primary cell wall formation by regulated targeting of cellulose-synthase complexes into the plasma membrane (Gutierrez et al., 2009).

Our results confirm the key role of cortical microtubules in xylem vessel differentiation in dodder shoots. Besides the supply and redistribution of water and nutrients throughout the rapidly elongating dodder seedling, xylem differentiation is also required for the formation of the functional connection between the parasite and the host plant necessary for the completion of life cycle of these parasitic plants after root-like structure degradation (Kaštier et al., 2017; Svubova et al., 2017). It has been found before that the differentiation of tracheal elements in *C. japonica* root-like structures differs from that in shoots (Lee et al., 2000). In *C. pentagona* seedlings, the conductive mesh is terminated close to the top of root-like structure and remains intact during its degradation (Sherman et al., 2008). Vascular elements are already formed in an embryo near the top of a root-like structure (Lyshede, 1986). Therefore, the differentiation of the tracheal elements in dodder shoots seems to be similar to non-parasitic plants.

### Microtubules and Actin Filaments in Dodder Cells After Treatments With Cytoskeletal Drugs

Taxol (paclitaxel) in plants can stabilize or over-polymerize cortical microtubules, resulting in club-like swelling of cells and trichome branching (Baluška et al., 1997; Mathur and Chua, 2000; Wani and Horwitz, 2014; Tian et al., 2015). Taxol and oryzalin effects on growth of both dodder species were more pronounced as compared to latrunculin B, cytochalasin D and jasplakinolide. Both shoot apex and root-like structure were swollen up to 7th day post-germination, and the nuclei of the epidermal cells were shifted from central positions to the cell peripheries, because taxol treatment disturbs cell elongation and promotes vacuolation (Bibikova et al., 1999).

The other microtubule-depolymerizing agent, dinitroaniline herbicide oryzalin, causes morphological changes similar to taxol such a club-like shoot swelling due to the disruption of cortical microtubules in shoot apical meristem cells. This is consistent with results of other authors on non-parasitic plants (Bartels and Hilton, 1973; Hoffman and Vaughn, 1994). However, swollen root hair-like trichomes of dodders because of taxol and oryzalin degraded, in contrast to the redirection of trichome growth and the initiation of their new branching sites in *Arabidopsis* (Mathur and Chua, 2000). The radial swelling of vestigial root cells was induced especially by oryzalin, taxol and latrunculin B treatments. In turn, the disturbance of actin filaments is related to the reduced cell elongation and expansion causing the “bonsai” phenotype of plants (Baluška et al., 2001; Mathur and Hülskamp, 2002). Actin filament polymerization and formation of G-actin complexes were challenged by macrolide compound latrunculin B (Coué et al., 1987; Spector et al., 1999; Baluška et al., 2001), leading to the inhibition of seedling growth of both *C. monogyna* and *C. europaea*, as well as the swelling of shoot apex and root-like structure. Baluška et al. (2001) also observed the severe growth retardation and thickening of plant organs, but no other significant morphological changes in latrunculin B-treated plants. This is supporting our observations. One possible explanation of cell elongation cessation is the perturbance of Golgi-derived vesicle trafficking of materials required for cell extension due to the F-actin cable disruption (Mathur and Hülskamp, 2002).

Both actin stabilizing drugs as well as those that interfere with actin polymerization result in the same dwarf phenotype (Mathur and Hülskamp, 2002). Indeed, cytochalasin D, which inhibits actin polymerization (Gibbon et al., 1999), and jasplakinolide, a commonly used actin filament stabilizing drug (Šamaj et al., 2002), reduced dodders’ growth and affected the morphology of shoot apex and root-like structure in the same manner as latrunculin B, which also prevents actin polymerization.

Finally, BDM, an inhibitor of myosin ATP-ase, did not have such a strong inhibitory effect on dodder seedling growth. It has been found that BDM treatment affected the distribution of myosin VIII, perturbed the interaction between microtubules and actin filaments, and increased the frequency of cortical preprophase bands (Šamaj et al., 2000). Moreover, BDM may affect the orientation of F-actin filaments and bundles, favoring the formation of more prominent transverse arrays (Hoffmann and Nebenführ, 2004; Cai et al., 2014), which we also observed in dodder shoots.

### The Eager Hands: Cytoskeleton of Dodder Prehaustoria

When dodder attaches to the host, newly formed lower haustorium (Lee and Lee, 1989), also called prehaustorium, enters the host tissue. Epidermal cells of the prehaustorium (called searching hyphae) start to elongate by tip growth, similarly to pollen tube, root hair or fungal hyphae, searching for the host xylem and phloem (Vaughn, 2002).

Previous anatomical studies showed that dodder prehaustorium has endogenous origin from a disk-like meristem (Lee and Lee, 1989; Svubova et al., 2017). In developing *C. europaea* prehaustorium several functional zones were found similarly to those of *C. japonica* (Lee, 2007). We have found that compressed cells (Lee and Lee, 1989) were mitotically active in *C. europaea*. Transverse arrays of cortical microtubules in digitate cells allow them to expand both laterally and longitudinally and switch into rapid cell elongation similarly to primary root apices (Baluška et al., 2001). At the same time, abundant actin filaments help to keep the nucleus at central position. Such organization of actin filaments and bundles in early stages of prehaustorium development is likely associated with active exocytosis of vesicles bearing de-esterified pectins, which ensure firm attachment of dodder shoot to the host prior to penetration (Estabrook and Yoder, 1998; Vaughn, 2002; Yoder and Scholes, 2010; Svubova et al., 2017). Reorganization of actin cytoskeleton during the penetration of prehaustorium to the host tissues could also support targeted secretion of various cell wall lytic enzymes, such as pectinases and cellulases (Srivastava et al., 1994; Vaughn, 2003).

Both microtubules and actin filaments are important for prehaustorium development in dodders. Taxol and oryzalin caused evident swelling of shoot apices and inhibition of seedling growth. Seedlings treated with the actin-targeted drugs and BDM were longer and less swollen, which is in accordance with the inhibition of seedling growth and morphological changes during *Cuscuta* germination at earlier developmental stages. Thus, different character of dodder response to microtubule- and actin microfilament-compromising chemicals might indicate the distinct roles of these cytoskeletal components in shoot apex elongation, bending toward the host, twisting and coiling around its stem as well as in prehaustorium formation. Direct function of mitotic microtubules in prehaustorium formation is likely related to cell divisions in disk-like meristem cells, and also in cells above the digitate ones, while cortical microtubule arrays support elongation of digitate cells. Switch of microtubule orientation is probably one of the mechanisms redirecting growth of epidermal cells toward the host.

**FIGURE 10:**
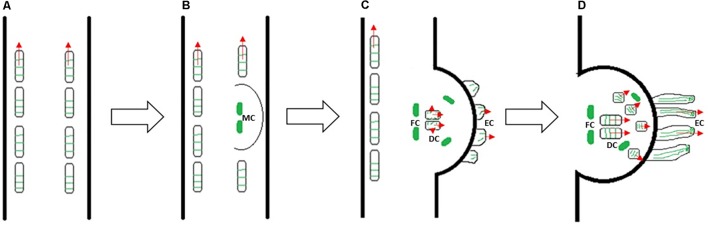
A model of *Cuscuta* prehaustorium initiation and growth (growth direction is indicated by red arrows), showing microtubule orientation (green) in cortical cells of shoot **(A)** and prehaustorium **(B–D)**. Transverse microtubules oriented perpendicularly to the cell expansion axis provide rapid elongation of *Cuscuta* seedling **(A)**. Dedifferentiation of cortex cells and formation of disk-like meristem (MC: meristematic cells) is essential for the development of fully functional haustorium at the site of parasite–host contact **(B)**. The orientation of microtubules changes in prehaustorial epidermal cells (EC), elongating digitate cells (DC), dividing file cells (FC) and cells above the digitate cells **(C)**. Reorientation of microtubules in cells above digitate cells and longitudinal alignment of microtubules in tip-growing epidermal cells of prehaustorium **(D)**.

Furthermore, elongating prehaustorium shows a high mitotic activity not only in file cells, but also in the cells positioned above the digitate ones (Svubova et al., 2017). Thus, cytoskeletal arrays in dodder cells contribute both to cell division and cell elongation in a same way as it has been observed in many non-parasitic plants. Our data suggest the developmental switch from transverse to longitudinal orientation of microtubules during prehaustorium formation, as depicted in **Figure 10**. We propose that tip-growing epidermal cells might be precursors of searching hyphae, which was previously assigned to digitate cells in *C. pentagona* (Alakonya et al., 2012).

After reaching host sieve elements a dodder searching hyphae changes to absorbing hyphae (Dawson et al., 1994; Vaughn, 2006). It will be also interesting to understand the mechanisms of reciprocal rearrangement of parasite and host cytoskeletons that most probably mimic the ones during plant–pathogen interaction (Takemoto and Hardham, 2004; Schmidt and Panstruga, 2007; Lapin and Van den Ackerveken, 2013). For instance, actin filaments of the host can be also involved in the response to the parasitic plants as a part of elicitor-triggered susceptibility, in a similar manner as against *Pseudomonas syringae* (Henty-Ridilla et al., 2013).

Generally, this is the first report about the organization of microtubules and actin cytoskeleton in parasitic plants during germination, without the contact with the host, but also during the formation of prehaustorium. It is shedding more light on dodder attachment to the host, and to the penetration mechanics. Both microtubules and actin filaments are present at early developmental stages of dodder seedlings in shoot apex and root-like structure cells. In root-like structure, a temporary anchorage organ, no mitotic arrays were found during early germination. Moreover, cortical microtubules and actin filaments in this organ start to degrade and cells undergo programmed senescence and plasmolysis at 5th–7th day post-germination. Practical impact of our results is a better understanding of the cellular and cytoskeletal rearrangements leading haustoria formation, which could be helpful in the development of efficient and low-cost control measures for *Cuscuta*. This is a challenge for plant pathologists, agronomists, and biotechnologists. A main remaining but quite difficult task will be to visualize the cytoskeleton in the haustorial bridge between parasite and host plant.

## Author Contributions

AB and YK developed concept and supervised this research. PK, YK, EP, and MM conducted the experiments. AB, EP, YK, PK, and JŠ analyzed and interpreted the data. EP and JŠ provided microscopic infrastructure. YK, PK, and AB wrote the manuscript with contribution of all co-authors.

## Conflict of Interest Statement

The authors declare that the research was conducted in the absence of any commercial or financial relationships that could be construed as a potential conflict of interest.

## Footnotes

1 https://imagej.net/Downloads
