# Supplementary material for: Cytoskeleton in the Parasitic Plant Cuscuta During Germination and Prehaustorium Formation
Source: Front Plant Sci. 2018 Jun 13;9:794. doi: 10.3389/fpls.2018.00794 (PMC6018488; doi:10.3389/fpls.2018.00794)
Supplement: Supplementary file 1 [file Data_Sheet_1.docx]

Supplementary Material

**Cytoskeleton in the parasitic plant *Cuscuta* during germination and prehaustorium formation**

Peter Kaštier^1+^, Yuliya A. Krasylenko^2,3^*^+^, Michaela Martinčová^1^, Emmanuel Panteris^4^, Jozef Šamaj^2^, Alžbeta Blehová^1^

^1^Department of Plant Physiology, Faculty of Natural Sciences, Comenius University in Bratislava, Bratislava, SK, Slovakia

^2^Department of Cell Biology, Centre of the Region Haná for Biotechnological and Agricultural Research, Olomouc, CZ, Czech Republic

^3^Department of Cell Biology and Biotechnology, Institute of Food Biotechnology and Genomics, Natl. Acad. Sci. of Ukraine, Kyiv, UA, Ukraine

^4^Department of Botany, School of Biology, Aristotle University of Thessaloniki, Thessaloniki, GR, Greece

*Author for correspondence:

Yuliya Krasylenko

[yuliya.krasylenko@upol.cz](mailto:yuliya.krasylenko@upol.cz)

^+^ these two authors contributed equally to this work

**
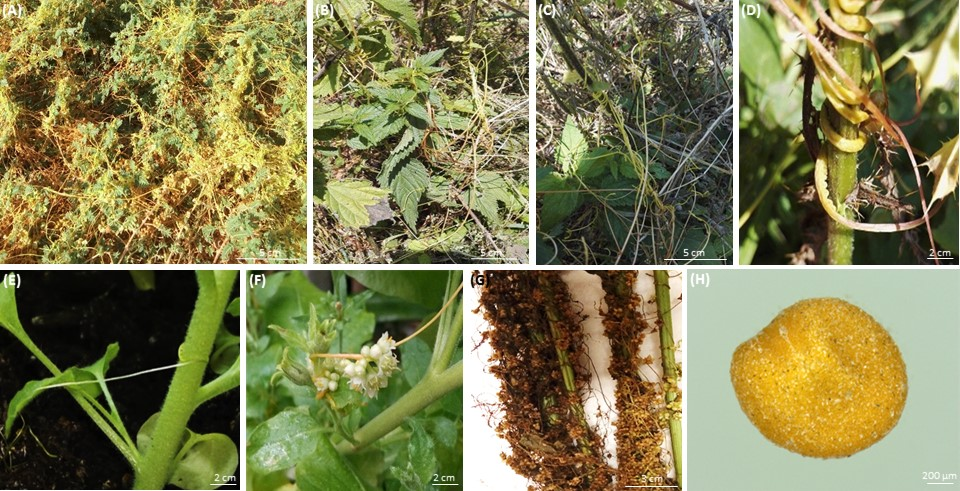
**

**Figure S1.** The European dodder (*C. europaea* L.) in nature and in a greenhouse: **(A)** mature parasite with fruits on its common host goat’s-head (*Tribulus terrestris* L.), Thessaloniki, Greece (40°35'34.3"N 22°58'20.1"E); **(B,C)** feeding parasite formed haustoria on common nettle (*Urtica dioica* L.), Ivanka pri Dunaji, Slovak Republic (48°11'35.7"N 17°14'23.7"E); **(D)** close-up of the haustoria on *U. dioica* stem; **(E)** parasite on tobacco (*Nicotiana benthamiana* Domin.) in a greenhouse; **(F)** flowers; **(G)** fruits; **(H)** seeds.


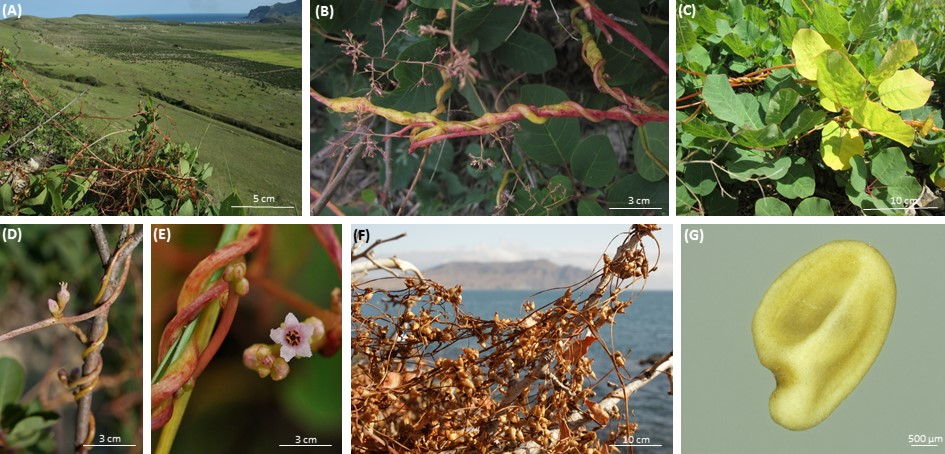


**Figure S2.** The Eastern dodder (*C. monogyna* Vahl.) in nature: **(A)** mature feeding parasite on the European smoketree (*Cotinus coggygria* (Scop.)), Uzyn-Syrt Hill, Koktebel environs, Crimean Peninsula (44°58'30.6"N 35°12'51.8"E); **(B)** self-parasitizing; **(C)** wilting of *C. coggygria* leaves as a result of parasitizing; **(D,E)** flowers at different developmental stages; **(F)** wilted parasite with seeds on pistachio (*Pistacia mutica* Fisch. et C.A.Mey.), Cape Alchak, Sudak environs, Crimean Peninsula (44°49'53.0"N 34°59'36.2"E); **(G)** seeds.


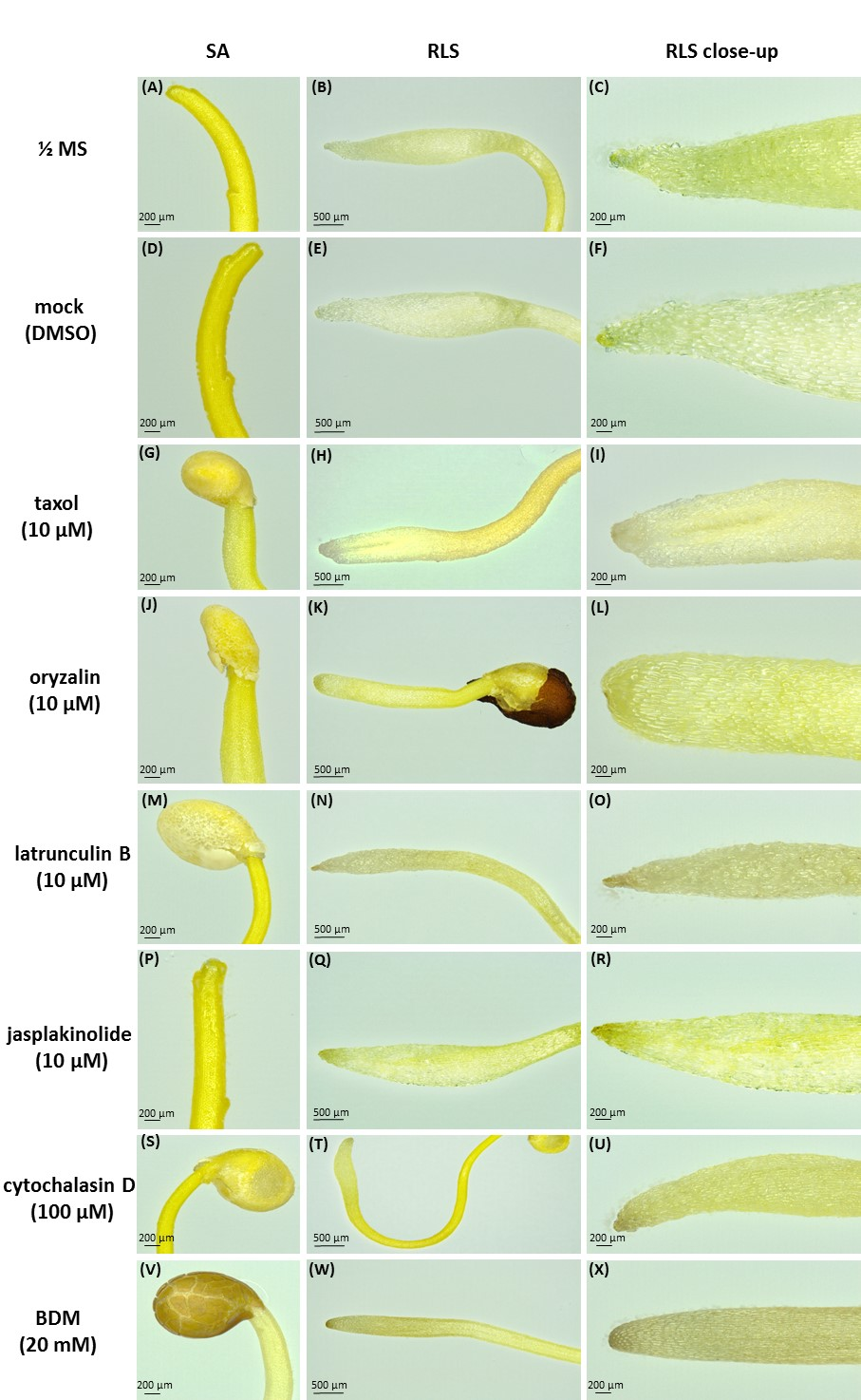


**Figure S3.** Effects of cytoskeleton drugs on 4 day-old *C. europaea* seedlings morphology: **(A–C)** ½ Murashige-Skoog medium; **(D–F)** mock (0.001% DMSO); **(G–I)** taxol (10 μM); **(J–L)** oryzalin (10 μM); **(M–O)** latrunculin B (10 μM); **(P–R)** jasplakinolide (10 μM); **(S–U)** cytochalasin D (100 μM); **(V–X)** BDM (20 mM). Abbreviations: SA – shoot apex; RLS – root-like structure.


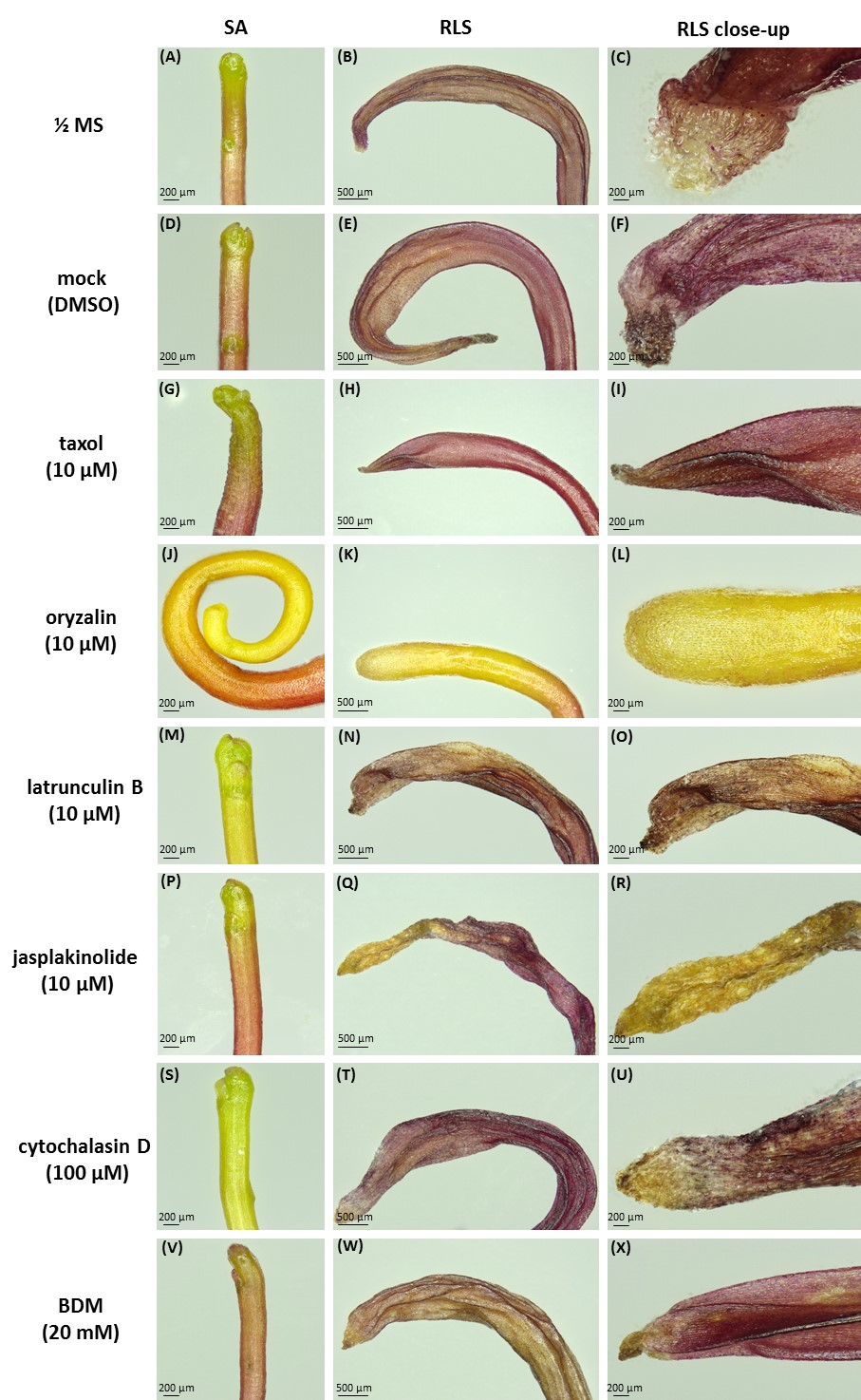


**Figure S4.** Effects of cytoskeleton drugs on 7 day-old *C. monogyna* seedlings morphology: **(A–C)** ½ Murashige- Skoog medium; **(D–F)** mock (0.001% DMSO); **(G–I)** taxol (10 μM); **(J–L)** oryzalin (10 μM); **(M–O)** latrunculin B (10 μM); **(P–R)** jasplakinolide (10 μM); **(S–U)** cytochalasin D (100 μM); **(V–X)** BDM (20 mM). Abbreviations: SA – shoot apex; RLS – root-like structure.


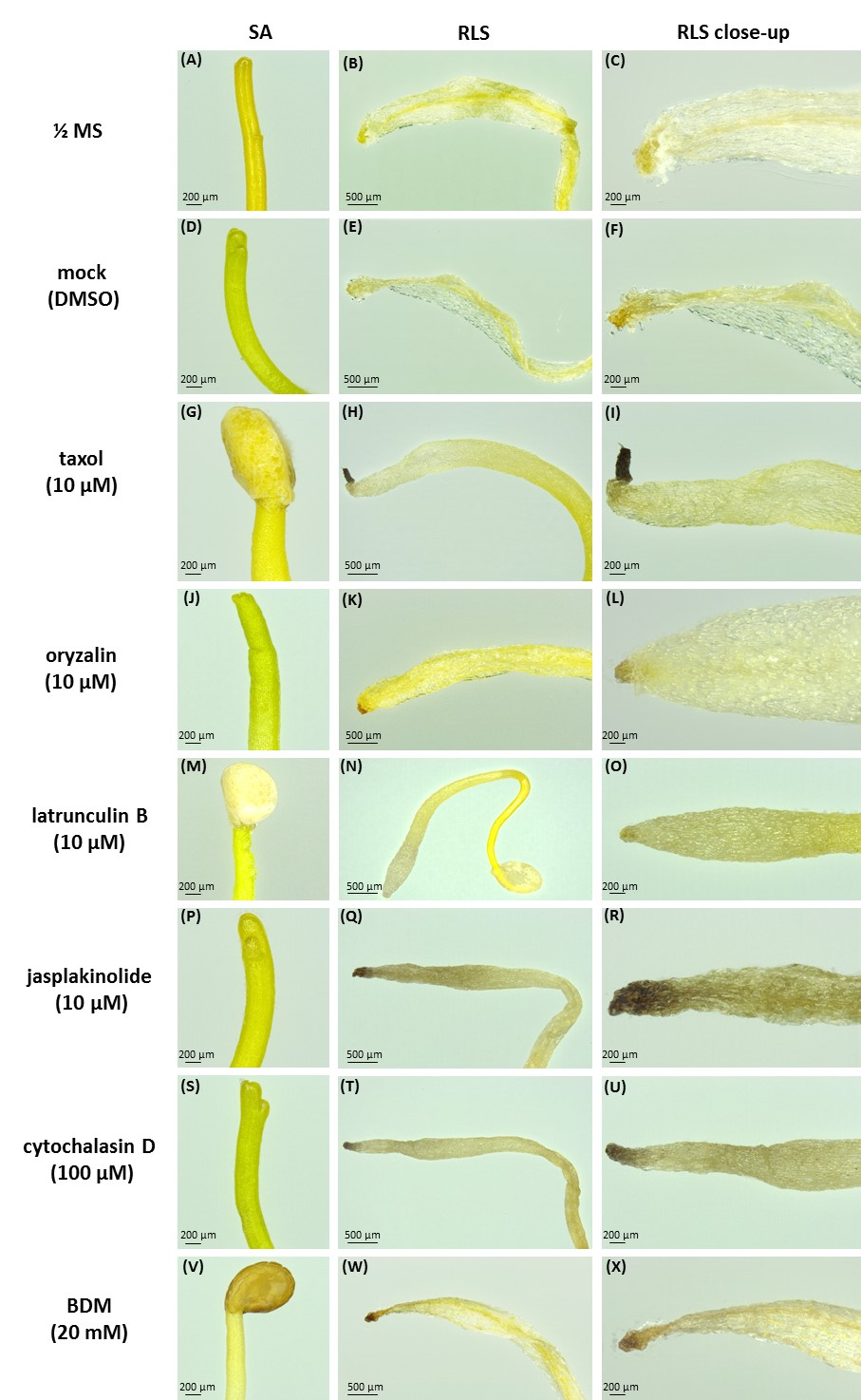


**Figure S5.** Effects of cytoskeleton drugs on 7 day-old *C. europaea* seedlings morphology: **(A–C)** ½ Murashige- Skoog medium; **(D–F)** mock (0.001% DMSO); **(G–I)** taxol (10 μM); **(J–L)** oryzalin (10 μM); **(M–O)** latrunculin B (10 μM); **(P–R)** jasplakinolide (10 μM); **(S–U)** cytochalasin D (100 μM); **(V–X)** BDM (20 mM). Abbreviations: SA – shoot apex; RLS – root-like structure.


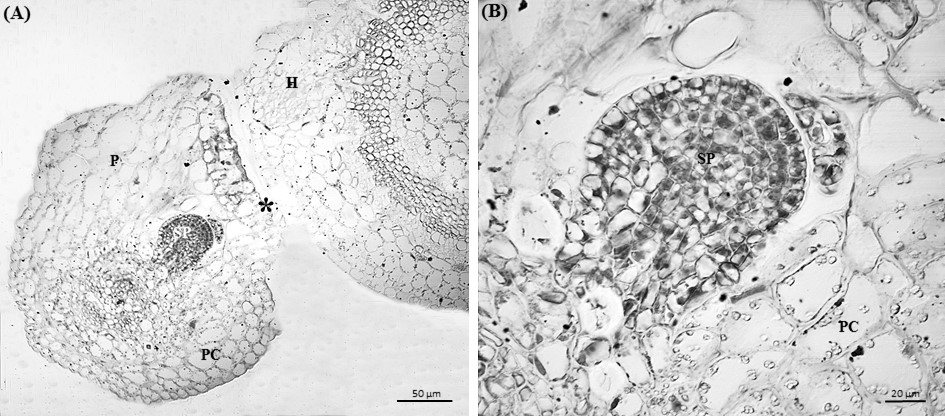


**Figure S6.** *C. europaea* shoot primordium formation after its contact with its host *N. benthamaiana*. Abbreviations: H – host; P – parasite; PC – cortical cells of parasitic shoot; SP – shoot primordium. Contact between parasitic and host plants is indicated by asterisk (*).
